# Advances in Extracting Bioactive Compounds from Food and Agricultural Waste and By-Products Using Natural Deep Eutectic Solvents: A Circular Economy Perspective

**DOI:** 10.3390/molecules29194717

**Published:** 2024-10-05

**Authors:** Petar Ristivojević, Maja Krstić Ristivojević, Dalibor Stanković, Ilija Cvijetić

**Affiliations:** 1 Department of Analytical Chemistry, Faculty of Chemistry, University of Belgrade, Studentski trg 12-16, 11158 Belgrade, Serbia; dalibors@chem.bg.ac.rs (D.S.); ilija@chem.bg.ac.rs (I.C.); 2 Department of Biochemistry, Faculty of Chemistry, University of Belgrade, Studentski trg 12-16, 11158 Belgrade, Serbia; krstic_maja@chem.bg.ac.rs

**Keywords:** circular economy, natural deep eutectic solvents (NADESs), food waste and by-products, bioactive compounds, extraction techniques

## Abstract

Due to the urgent need for a transition to sustainable, zero-waste green technology, the extraction of bioactives from food and agricultural by-products and waste has garnered increasing interest. Traditional extraction techniques often involve using organic solvents, which are associated with environmental and health risks. Natural deep eutectic solvents (NADESs) have emerged as a promising green alternative, offering advantages such as low toxicity, biodegradability, and the ability to dissolve a wide range of biomolecules. This review provides a comprehensive overview of recent trends in the application of NADESs for extracting bioactive compounds from sustainable sources. The review explains the composition and principles of preparation and highlights various applications of NADESs in extracting different classes of bioactive compounds, emphasizing their potential to revolutionize extraction processes. By summarizing the latest advancements and trends, this review aims to support research and industrial applications of NADESs, promoting more sustainable and efficient extraction methods in the food and agricultural sectors.

## 1. Introduction

The processing of millions of tons of food each year in Europe results in substantial losses and waste, with around 13% lost from harvest to retail and an additional 17% wasted globally by households, services, and the retail sector [1]. Food production, which accounts for nearly half of global waste, offers a significant potential for a transition to a circular economy. Both industry and households generate considerable amounts of organic by-products and waste, raising concerns about economic and environmental impacts, including financial costs, water and land pollution, biodiversity loss, and greenhouse gas (GHG) emissions [2]. 

Annually, food waste contributes to 8% of human-induced GHG emissions and consumes 45 trillion gallons of water globally, representing 24% of total agricultural water use. Within the European Union, 58 million tons of food waste are generated each year [3], which corresponds to 131 kg per person and a value of 132 billion euros [1]. Traditional methods of dealing with food waste, such as producing animal feed, fertilizers, landfilling, and formal disposal, face limitations due to legal restrictions, ecological issues (GHG emissions), and high costs. Therefore, valorizing food waste and by-products offers alternatives to these traditional waste management methods [4]. By focusing on minimizing environmental exposure and health hazards, efficient, cost-effective, and ecologically sound food waste and by-product management aims to utilize non-edible biomass in high-value bio-based products, thereby maximizing the value of the initial biomass as a feedstock [5]. 

**Figure 1:**
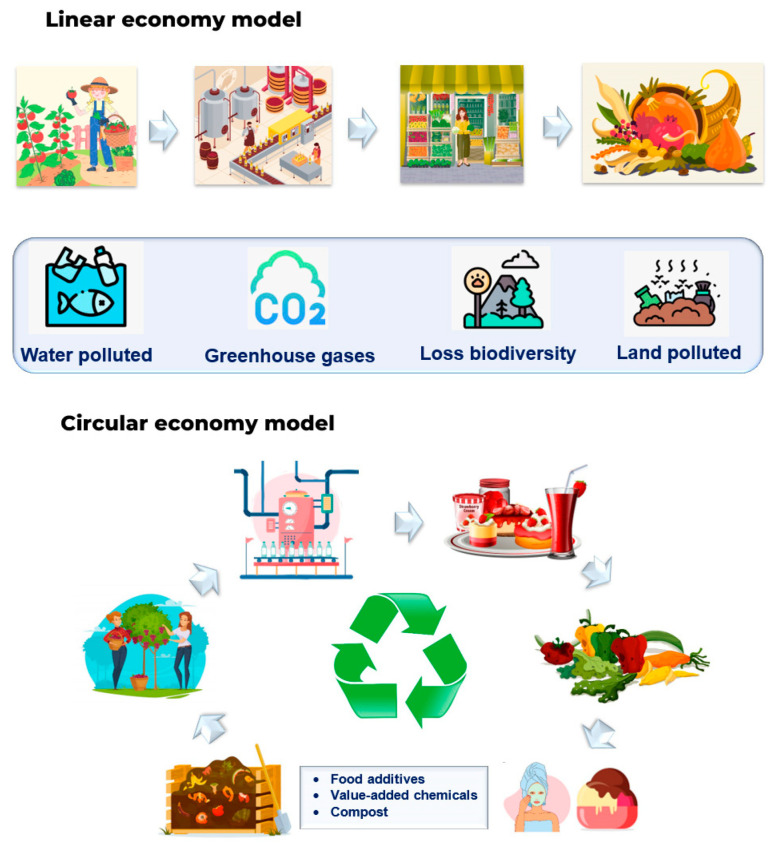
Linear and circular economy models in the food industry.

Food and agricultural by-products, like peels, seeds, leaves, and stems, are often discarded as waste, even though they are rich in valuable bioactive compounds such as polyphenols, terpenes, flavonoids, alkaloids, and vitamins, which have important health benefits. These compounds can be used in the pharmaceutical, nutraceutical, and cosmetic industries [6]. Efficient extraction of these compounds adds value to agricultural waste and supports circular economy by promoting resource efficiency and sustainability [7]. Utilizing these by-products for bioactives not only reduces the environmental impact of waste disposal but also opens up new economic opportunities. For instance, turning waste into high-value food products can promote the development of sustainable industries and reduce the need for synthetic additives. Moreover, this approach encourages more sustainable agricultural practices, as farmers and food processors can generate additional income by selling by-products for the extraction of bioactives (Figure 1).

To improve efficiency and reduce the environmental impact of traditional extraction methods, a new generation of solvents called natural deep eutectic solvents (NADESs) has emerged as a viable, green, and sustainable alternative [8,9,10]. NADESs are created by mixing two or more natural compounds, typically involving a hydrogen bond donor and a hydrogen bond acceptor in a certain stoichiometric ratio [11]. These solvents are known for their low toxicity [12,13,14,15], biodegradability, stability [14], and tunable physicochemical properties [16], making them particularly suitable for extracting a wide range of target analytes. NADESs have the unique ability to dissolve a wide range of compounds, from small molecules to biomacromolecules, and are compatible with biological systems [17,18]. Recently, NADESs with a high water content, known as aqueous acidic solvents [18], have been recognized as effective solvents, leading to the proposal of the new term “Mixtures based on Natural Compounds—MINACs“ [19]. 

Recent studies have demonstrated that NADESs can enhance the extraction yield and efficiency of various bioactive compounds. They have shown that NADESs can outperform traditional solvents in terms of extraction efficiency, selectivity, and environmental impact. Moreover, NADES can simplify the extraction process by reducing the number of steps required. Being biodegradable and easily synthesized from renewable resources with low energy requirements, NADESs significantly reduce environmental impact compared to conventional solvents. Their health and safety benefits also make them ideal for applications in the food, pharmaceutical, and cosmetic industries [20,21]. 

Although the use of NADESs in extraction processes is still a relatively new field, it is rapidly gaining attention. An increasing body of literature explores various aspects of NADESs, from their fundamental properties to practical applications [22,23]. This review aims to provide a comprehensive overview of the recent trends in the extraction of bioactive compounds from fruits, vegetables, and their by-products and waste using NADESs. The paper will discuss the preparation, cost, and advantages of NADESs compared to conventional solvents, as well as their role in promoting a circular economy.

## 2. Natural Deep Eutectic Solvents: Composition and Preparation Methods

Three main features characterize NADESs: (i) they are a type of deep eutectic solvent (DES), (ii) their constituents are naturally occurring compounds, and (iii) unlike pure ingredients, which have a fixed melting point under constant pressure, NADES mixtures have a melting point that extends over a temperature range [24]. Moreover, they have a significantly lower melting point of NADES than its components, and considerable negative deviations from ideal melting points originate from strong intermolecular interactions, including hydrogen bonds, van der Waals forces, and ionic bonding. These interactions create stable mixtures that require less energy for the transition from solid to liquid, resulting in lower melting points and increased stability [25,26].

There are 127 scientific articles in the Scopus database (2011–2024) that include “Deep Eutectic Solvents”, “Natural Deep Eutectic Solvents”, or “NADES” in their titles, abstracts, or keywords, focusing on the extraction of bioactive compounds from natural resources. The composition of NADES varies significantly: 30% of the papers focus on choline chloride (ChCl) as a component, 17% on urea (U), 13% on glycine (Gly), 9% on lactic acid (LA), 9% on betaine (B), and 8% on glucose (Glu). ChCl is the most frequently used hydrogen bond acceptor (HBA), featured in 29% of the studies. 

**Figure 2:**
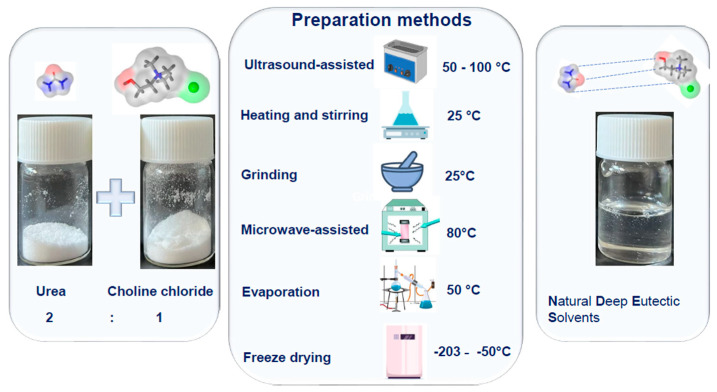
Preparation methods of natural deep eutectic solvents.

NADESs are typically synthesized using six physical methods: heating and stirring, freeze-drying, evaporation, grinding, ultrasound-assisted synthesis (UAS), and microwave-assisted synthesis (MAS) (Figure 2). In the heating and stirring method, each NADES component is accurately weighed and transferred to a beaker. The mixture is then heated to a temperature between 50 and 100 °C and stirred with a magnetic stirrer on a heating plate until it becomes a viscous, transparent liquid [27,28]. For the freeze-drying method, components are weighed, mixed with a specific molar ratio of water, and then freeze-dried at temperatures between 77 K and 253 K to sublimate the water and produce pure NADES [29]. In the evaporation method, the components are dissolved in water, and the mixture is then evaporated using a rotary evaporator. The NADESs are then stored in a desiccator until they reach a constant weight. The grinding method involves mixing and grinding the components in a mortar and pestle at room temperature until a homogeneous liquid is formed [30]. 

For ultrasound-assisted synthesis (UAS), the mixture is first homogenized in a vortex for about 1 min, then treated in an ultrasonic bath for 30 min, followed by another round of homogenization and 15 min of ultrasonic bath treatment before storing the NADES in a desiccator at room temperature. Microwave-assisted synthesis (MAS) involves homogenizing the mixture in a vortex for about 1 min, followed by treatment in a microwave reactor at 850 W and 600 rpm for 45 min [31]. Among these methods, freeze-drying, evaporation, and grinding are straightforward to perform, while UAS and MAS are faster and more efficient. This is because microwave radiation interacts with materials, causing dipole rotation and molecular collisions, leading to dielectric heating that shortens synthesis time. The cavitation effect from ultrasonic waves also promotes interaction between the HBD and HBA components. The heating and stirring methods are the most commonly used for preparing NADES because it is cost-effective, easy to operate, and allows for simple regulation of conditions, which is crucial for thermally unstable components [32].

## 3. Properties and Applications of NADES

Conventional solvents are typically toxic and harmful to the environment, with their high volatility contributing to both air and water pollution. Furthermore, organic solvents often exhibit low biodegradability, leading to environmental persistence. Organic solvents also present significant safety hazards due to their flammability and can leave toxic residues in final products, necessitating additional purification steps—an issue largely mitigated by the use of NADESs [9,11,26,27]. Moreover, organic solvents are predominantly petroleum-based, making them less sustainable and often more expensive, whereas NADESs are derived from renewable resources, fully aligning with the principles of green chemistry. Furthermore, regulatory restrictions on organic solvents, particularly in food and cosmetic industries, are stricter due to health concerns, whereas NADESs face fewer regulatory challenges, offering greater flexibility in product development. NADESs present significant advantages over traditional organic solvents, particularly in terms of safety, biodegradability, and sustainability. Unlike conventional solvents, which often pose health risks and environmental challenges, NADESs are typically derived from natural components and exhibit lower toxicity profiles [9,11,26]. The promising results obtained from various studies suggest that NADESs can enhance extraction efficiency and selectivity for bioactive compounds, making them ideal candidates for green chemistry applications. Unlike conventional organic solvents, NADESs are composed of plant metabolites such as sugars, amino acids, and organic acids. This composition makes them biodegradable and generally safer for both human health and the environment. The biodegradability of NADESs is a critical factor for ecological safety, with many NADESs being more than 60% biodegradable, although some studies have shown variability in this regard [33]. 

The effectiveness of NADESs as extractants is largely due to their extensive hydrogen bonding network, which provides excellent solubilization and stabilization capabilities for bioactives. These solvents have been reported to effectively extract a variety of compounds from food products, often utilizing eco-friendly UAS and MAS methods to enhance extraction efficiency. Notably, studies have demonstrated that NADES can prevent the photodegradation of curcumin, significantly improving its stability during storage [34]. Similarly, carthamin is twice as stable in NADES at 60 °C compared to water and five times more stable at 40 °C than at 60 °C, emphasizing the protective molecular interactions between sugary NADESs and bioactives against degradation caused by heat, light, and aging. NADESs have proven to be superior solvents for curcuminoids, enhancing their stability and antioxidant activity when composed of organic acids and sugars rather than traditional organic solvents. 

NADESs also function as green flavor enhancers thanks to their non-toxicity, low cost, and safe consumption profile. They facilitate the Maillard reaction, a key process that imparts unique food flavors. Research by Kranz et al. demonstrated that NADES could accelerate the formation of Maillard-derived taste regulators, with sugar-based NADES showing the most significant promotion [35]. Further studies indicated that NADESs composed of cysteine and those in alkaline conditions enhanced the production of thiamine-derived taste regulators, with yield rates dependent on temperature and pH levels [36].

In addition to their extraction and flavor-enhancing capabilities, NADESs show great promise as cryoprotectants, preventing protein denaturation and lipid oxidation during the frozen storage of meat products. They inhibit ice crystal formation and protein aggregation, reducing the number of ice crystals and minimizing ice crystal damage in cells during freezing, which is crucial for cell survival while also prolonging the shelf life of meat products [28]. Furthermore, NADES can serve as an antifreeze for food contact interfaces, enhancing the anti-frosting and deicing abilities of coated substrates under extreme conditions. The inclusion of proline, a natural antifreeze agent, in NADES formulations enhances their protective capabilities, making them a viable, green, and safe option for cryoprotection in the frozen food industry [37]. 

NADESs also exhibit strong antioxidant activity, particularly those based on organic acids, which is essential for improving the stability and shelf life of foods. Additionally, NADESs demonstrate significant antibacterial properties, particularly against Gram-negative bacteria, due to the interaction of their components with bacterial cell walls, leading to cell damage [38,39,40]. The toxicity of NADESs varies, with some showing low cytotoxicity and minimal impact on wheat seed germination, while others can inhibit cell metabolic activity [37]. 

Moreover, NADESs have been incorporated into food films as plasticizers, significantly improving the mechanical properties, elasticity, tensile strength, and water absorption performance of chitosan films. This incorporation not only enhances the quality of synthetic foods but also contributes to environmental sustainability by reducing the waste produced by traditional packaging materials [41]. 

The polarity of NADESs, which varies from highly hydrophilic to hydrophobic, impacts their ability to dissolve bioactives. Certain compositions, such as MA and choline chloride-based NADES, exhibit the highest polarity [42]. However, their high viscosity, which reduces the extraction efficiency, can be reduced by heating or diluting with water [31]. Overall, NADESs have diverse applications and properties, ranging from their role as effective extractants and flavor enhancers to their use in biodegradable films and cryoprotectants. Their ability to enhance the stability, safety, and quality of food products while also offering environmental benefits makes them a promising area for continued research and development [43].

## 4. Cost of NADES Solvents

The cost of natural deep eutectic solvents (NADESs) and their components is a crucial factor in their adoption and scalability for extracting target molecules. NADESs are typically made from readily available and inexpensive natural substances such as amino acids, organic acids, sugars, fatty acids, terpenes, and other plant metabolites. The cost of these individual components is generally low, as they are commonly used in the food and pharmaceutical industries [44]. For instance, compounds like CA, LA, Glu, and ChCl are widely used and economically accessible. 

The overall cost of NADES is influenced by factors such as the purity and source of the chemicals, the scale of production, and the specific formulation used [45]. While the initial investment in developing and optimizing technology for the preparation of NADES on an industrial scale for specific applications might be higher, the long-term benefits and cost savings from reduced solvent use, safer handling, and lower environmental impact make them economically attractive. Furthermore, as research advances and production methods improve, the cost of NADESs is expected to decrease, making them even more competitive with traditional solvents [46]. 

In summary, while the cost of NADES solvents and their components is relatively low due to the availability of natural raw materials, the economic advantages extend beyond mere component costs [47,48]. The overall savings in environmental and health-related expenses, coupled with their sustainability benefits, position NADES as a cost-effective solution for the extraction of bioactive from fruit and vegetable waste.

## 5. Solubility Challenges of NADES and the Role of Co-Solvents

NADESs are highly valued for their environmental friendliness and capacity to extract bioactives without the use of harmful chemicals. However, a significant limitation of NADESs lies in their solubility behavior. Specifically, NADESs have a propensity to form aggregates, which can substantially restrict the solubility of certain solutes. Previous research has highlighted how these aggregates, intrinsic to the NADES structure, can hinder their solubilization efficiency. For instance, studies on the solubility of lignin and curcumin have demonstrated that aggregation of NADES components impairs efficient solubilization. A deep eutectic solvent combined with ethanol was developed for the pretreatment of *Broussonetia papyrifera* to efficiently extract lignin and enhance subsequent enzymatic hydrolysis [49]. Further, research by Kunz’s team suggests that a NADES–ethanol mixture may enhance curcumin solubility by reducing aggregation, although this remains a hypothesis that requires further confirmation [50]. Preliminary results indicate that introducing co-solvents like ethanol can potentially create a more homogeneous solvent system, improving the performance of NADESs for challenging solutes. The addition of ethanol appears to disrupt the internal aggregation within NADESs, which may increase the solubility of hydrophobic or poorly soluble compounds and improve extraction efficiency. However, as the work is ongoing and complex, further studies are needed to substantiate these claims [51]. Thus, while NADESs offer numerous advantages, their solubility limitations may require optimization through solvent blending. 

## 6. Toxicity of NADES

Natural deep eutectic solvents (NADESs) are generally recognized as safer alternatives to conventional organic solvents, but their toxicity profiles vary significantly depending on their composition. The toxicity of NADES can be influenced by hydrophobic constituents such as thymol (Ty), resorcinol, and menthol (Me), which require careful evaluation. While NADESs may reduce overall toxicity compared to individual solvents, certain combinations can exhibit synergistic effects that might increase toxicity. Additionally, assessing the biodegradability and environmental persistence of NADESs is crucial, as the accumulation of less biodegradable components can pose ecological risks. Regulatory compliance in food and pharmaceutical applications necessitates comprehensive toxicological studies to establish safety for human health and environmental exposure. A more rigorous scientific approach is needed to compare the toxicity of NADESs with their constituent compounds, as much of the existing literature reports only experimental observations without thorough discussions of their relative advantages and disadvantages. References supporting this discussion include [46,52,53,54], which highlight the potential and limitations of NADESs in various applications.

Hydrophobic NADESs, such as those containing Ty, resorcinol, and Me, present significant toxicity concerns due to the known adverse effects of their components. Ty can be toxic to the liver and kidneys, resorcinol is a skin irritant that may disrupt thyroid function, and Me is neurotoxic at high doses. Additionally, the toxicity of the NADESs themselves can pose risks, as the interactions between the components may lead to unexpected effects not seen with individual substances. The use of these solvents in industries, particularly cosmetics, often lacks rigorous scientific backing, serving more as a marketing tool than a thoroughly validated solution. While some NADESs, such as those involving ChCl and sugars, have been applied in cosmetics for extraction purposes, few studies provide clear scientific comparisons to individual components, making safety and efficacy claims weak. In principle, NADES may improve extraction due to synergistic solvation, stabilization of extracts, and reduced volatility compared to their components [55,56]. Nevertheless, there is a shortage of detailed mechanistic studies regarding hydrophobic NADESs and comparisons with conventional solvents to strongly substantiate these claims.

## 7. Mechanism of Extraction of Bioactives Using NADES

**Figure 3:**
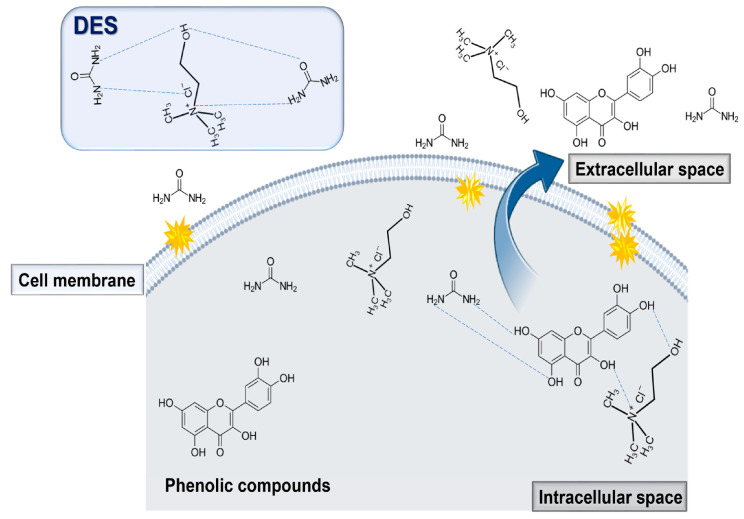
Extraction of phenolic compounds from natural sources.

The extraction of phenolic compounds from natural sources using NADES involves several key mechanisms, as illustrated in Figure 3. These solvents effectively solubilize phenolic compounds through hydrogen bonding and van der Waals interactions, owing to their high polarity and strong solubilizing power. Additionally, NADESs facilitate the disruption of cellular structures, including cell walls, which aids the release of intracellular phenolics. 

The interactions between NADES and phenolic compounds are complex, including mechanisms such as hydrogen bonding, van der Waals, and acid-base interactions. For instance, the hydroxyl groups in phenolics can form hydrogen bonds with NADES components, thereby enhancing the extraction efficiency. Additionally, some NADES components may form complexes with phenolics, which can further improve their solubility and increase extraction yields [49]. 

## 8. Extraction of Bioactive Compounds from Fruit Waste and By-Products 

The extraction of bioactives from fruit by-products and waste has emerged as a crucial area of research, driven by the need for sustainable and efficient utilization of natural resources. Fruits, particularly their by-products and waste, are rich in fibers, vitamins, and bioactive compounds, which have significant health benefits. However, these valuable compounds often remain underutilized due to the limitations of conventional extraction methods. 

NADESs have the potential to overcome these limitations by enhancing the solubility and stability of bioactives, enabling more efficient and effective extraction. This not only maximizes the value derived from fruit waste but also supports the development of functional foods, nutraceuticals, and cosmetic products. The use of NADES in extracting bioactives from fruit waste represents a significant advancement in sustainable technology. By leveraging the unique properties of NADES, the extraction process becomes more efficient, safer, and environmentally friendly, paving the way for innovative applications across various industries. 

Numerous studies have demonstrated the efficacy of NADES in enhancing extraction yields, improving selectivity, and preserving the bioactivity of extracted compounds. Table 1 provides an overview of the extraction processes for phenolic compounds from fruit and vegetable waste and by-products [46,47,48].

De Oliveira et al. (2013) [57] investigated the extraction of flavonoids from citrus peels (citrus pomace with and without pectin) using NADES composed of choline chloride, betaine, Gly, and various carboxyl acids. They found that mixtures of betaine/LA and choline chloride/Gly were the most effective, yielding a higher total phenolic content compared to conventional solvents. HPLC analysis under optimized conditions revealed that DES-based UAE extracts contained 14 phenolic compounds, with high levels of vicenin-2 (17.6 mg/g) and orientin (23.6 mg/g). 

In another study, Mirella R.V. Bertolo and colleagues utilized a mixture of ChCl and CA to extract phenolic compounds from pomegranate peels. They characterized five choline chloride-based NADESs, containing Glc, sucrose, Gly, LA, and CA, and tested for the recovery of phenolics from pomegranate peel. Among these, the combination of ChCl and LA proved to be the most suitable for extracting total phenolic content from pomegranate peel (4.14 mg EAG/mL) and the ethanolic solution (3.55 mg EAG/mL) [58].

An ultrasound-assisted method has been developed for the environmentally friendly extraction of phenolic compounds from blueberry leaves using NADESs. After the screening of multiple solvents, the best extraction efficiencies in terms of total phenolic content (TPC) and antioxidant activity were provided by mixtures of LA, Na Ace, and water (3:1:2), and ChCl and OxA (1:1.) LA and ChCl provided superior performance for the recovery of phenolic compounds (1.6-fold and 2.2-fold greater efficacy, respectively) and antioxidant compounds (1.6-fold and 2.8- fold greater efficacy, respectively) [59]. 

Extracts rich in phenols and flavonoids obtained from orange peel waste using NADES were monitored for stability over 30 days. The most effective solvents were LA:Glu and L-Pro:MA. The extraction process with these NADESs was optimized, and the results showed that %NADES, solid:liquid ratio, and extraction time significantly influenced the extraction efficiency in terms of TPC and total flavonoid content (TFC). The most promising solvents identified were LA:Glu, which achieved an extraction yield of 1932 ± 7.83 mg GAE/100 g dw for TPC and 82.7 ± 3.0 mg/100 g dw for TFC. Similarly, LA:MA exhibited even higher yields, with 2164 ± 5.17 mg GAE/100 g dw for TPC and 97.0 ± 1.65 mg/100 g dw for TFC. These results highlight the efficiency of both solvents in extracting high concentrations of polyphenols and flavonoids [60].

The peels from different Croatian cultivars of Citrus reticulata Blanco mandarin (Zorica rana, Chahara, Okitsu, Kuno) were extracted using 15 different choline chloride-based DESs, with 20% water added. The screening results indicated that choline chloride:acetamide (1:2) was the most effective for hesperidin extraction. The choline chloride: acetamide (1:2) provided the most efficient hesperidin extraction (112.14 mg/g of plant). [61]. In another study, a choline chloride-based DES combined with Gly was used to extract phenolics from citrus peels. Response surface methodology (RSM) was employed to optimize the extraction parameters, namely, the water concentration (CW), the liquid-to-solid ratio (RL/S), and the duration of the extraction (t), focusing on TPC and radical scavenging activity (RSA). A validated RP-UPLC–ESI–QTOF-MS method showed good precision (RSD% < 9.8%). It quantified 10 target compounds and tentatively identified 13 additional compounds. Ten compounds were determined and quantified through target screening, as well as 4foursuspects, and nine non-target compounds were tentatively identified in the extracts using suspect and non-target screening, respectively. [62]. 

Apple pomace extracts obtained through non-conventional green extraction methods and DES systems were also evaluated. The antioxidant capacity was measured using DPPH and FRAP assays, and the ability of extracts to stimulate insulin secretion from pancreatic beta cells was assessed. The effects of the extracts on cell viability and insulin secretion were evaluated using the BRIN-BD11 cell line. Extracts obtained with ChCl:EG (1:4) demonstrated a significantly higher antioxidant capacity in the DPPH assay, showing 80.1% inhibition compared to just 11.3% for the control. Although these extracts were less effective at stimulating insulin secretion than those obtained using classical systems, they demonstrated the potential of DES systems for extracting bioactive compounds with beneficial properties for metabolic health [63].

An eco-friendly method using pulsed electric field-assisted extraction (PEF-AE) with DESs was applied to recover flavonoids from noni pomace. The study identified choline chloride/EG and choline chloride/OxA as the optimal extractants, significantly improving the recovery of rutin and quercetin compared to conventional methods. After response surface methodology optimization, the highest extraction amounts of rutin and quercetin were 16.21 mg/g and 19.85 mg/g, respectively. This approach is efficient, selective, and sustainable, demonstrating the potential of combining PEF with DESs for extracting flavone aglycones from noni-pomace waste [64]. 

Li and coworkers developed a green and efficient approach based on pulsed electric field (PEF) in combination with DES to enhance the antiglycation ability of noni flavonoids by modulating their proportions of flavone glycosides and aglycones. Quercetin levels increased with longer PEF treatments (2 kV/cm, 30 μs, 10 Hz; DES: 100% choline chloride/OxA). After 4000 treatments, the rutin:quercetin molar ratio shifted from 0.92:0.08 to 0.09:0.91 [65]. A sustainable method combining HVED and DES for extracting proteins and polyphenols from pomegranate seeds was developed. HVED pretreatment, which disintegrated 86% of cells at 160 kJ/kg energy input, outperformed ultrasounds, leading to 70% protein and 78% polyphenol release. Solid–liquid extraction using five DESs showed enhanced diffusivity for both, with proteins extracted at higher rates, though NaOH yielded greater overall amounts [66]. Similarly, El Kantar et al. (2019) investigated the green extraction of polyphenols from grapefruit peels using HVED, DES, and aqueous Gly. They found that 20% aqueous Gly or DES significantly reduced the HVED energy input while maintaining polyphenol diffusivity and improving the solubility of naringin. The HVED energy input was reduced by 6-fold when solid–liquid extraction was performed in 20% (*w*/*v*) aqueous Gly or DES (LA: Glc) instead of water. Polyphenol diffusivity (4 × 10^−11^ m^2^/s) was the same in water at 218 kJ/kg and in aqueous Gly at 36 kJ/kg after pretreatment [67]. Another study investigated the recovery of phenolic compounds from ripe mango (*Mangifera indica* L.) peel using a microwave-assisted DES extraction method, with LA/sodium acetate/water (3:1:4) as the extractant. The optimized conditions resulted in a significant recovery of TPC, with HPLC analysis identifying mangiferin as the prominent phenolic compound. The optimized conditions were 436.45 W, 19.66 min, and a liquid-to-solid ratio of 59.82 mL/g. Under these conditions, total phenolic content recovery was 56.17 mg GAE/g dw, FRAP was 683.27 µmol AAE/g dw, and DPPH scavenging activity reached 82.64%. Additionally, the oxidative stability of sunflower oil nearly doubled with the addition of purified mango peel extracts [68].

Further research examined the use of seven NADESs to extract valuable compounds from orange peel waste. Among these, choline-based NADES with EG (ChEg50) was particularly effective due to its superior enantioselectivity in kinetic resolution, enzyme stability, and ability to extract D-limonene, polyphenols, and proteins. The ChEg50 also showed a satisfactory capacity to extract D-limonene (0.5 mg gFW^−1^), polyphenols (45.7 mg gFW^−1^), and proteins (7.7 mg gFW^−1^) from the peel [69].

Panić et al. developed an eco-friendly method for extracting anthocyanins from grape pomace using a combination of ChCl and CA, along with multimode-microwave and low-frequency-ultrasound irradiation. Optimal conditions were achieved with simultaneous ultrasound/microwave-assisted extraction (UMAE), yielding 1.77 mg/g dw^−1^ of anthocyanins. The method also allowed efficient recovery and recycling of NADES in a scaled-up half-liter batch process [70].

Additionally, a sustainable and green extraction method for phenolic compounds from red grape pomace was optimized using NADESs such as betaine combined with CA, U, and EG, specifically for cosmeceutical applications. The B-CA combination showed the best flavonoid permeation and antioxidant activity in human keratinocytes, making it a promising cosmetic ingredient for reducing oxidative stress and inflammation, which are key factors in skin aging. The optimized conditions were 436.45 W, 19.66 min, and a liquid-to-solid ratio of 59.82 mL/g. Under these conditions, total phenolic content recovery was 56.17 mg GAE/g dw, FRAP was 683.27 µmol AAE/g dw, and DPPH scavenging activity reached 82.64% [71].

An industrially suitable extraction process for sustainable waste disposal in wine production was developed, focusing on extracting bioactives from Graševina grape pomace using 45 environmentally friendly DESs. The extract obtained with BGlc contained the highest total polyphenols yield (26.07 mg g dw^−1^ of pomace), analogous to that obtained with EtOH as the reference solvent (26.06 mg g dw^−1^ of pomace). Both computational and experimental results confirmed the effectiveness of COSMOtherm in selecting the optimal DES, enabling the preparation of safe extracts for potential use in the cosmetic industry [72]. In related research, Dabetić et al. demonstrated that DES, particularly a combination of ChCland CA, effectively extracted phenolic antioxidants from ten grape varieties. The highest (+)-catechin content was found in ‘Župljanka’ seeds (15.587 mg g^−1^ DW with acidified aqueous ethanol and 10.197 mg g^−1^ DW with ChCit). Their findings showed that grape seeds exhibited higher antioxidant capacity compared to grape skins, with DES often outperforming conventional solvents in both extraction efficiency and antioxidant activity [73].

Further advances in the field include the optimization of ultrasound-assisted extraction (UAE) using NADES, particularly ChCl–LA (1:2), for isolating non-extractable polyphenols from mangosteen peel as potential antioxidants. The study included a green extraction method using UAE with ChCl–LA for isolating antioxidants from mangosteen peel. The process yielded high proanthocyanidin content with low cytotoxicity, suggesting its potential for sustainable applications in various industries [74]. Similarly, polyphenols were extracted from pomegranate peels using solid–liquid, ultrasound, and Ired-Irrad^®^ infrared extraction in combination with DES as green alternatives. Among these methods, the infrared technique combined with DES yielded the highest polyphenol concentration (152 mg/g DM) and exhibited superior antioxidant, antiradical, antimicrobial, and antifungal activities, demonstrating its high selectivity in polyphenol extraction [75].

In another study, ten different NADESs were utilized for the extraction of procyanidins and anthocyanins from cranberry pomace using UAE. The combination of choline chloride, betaine hydrochloride, and levulinic acid in a 1:1:2 ratio with 32 mL water/100 mL NADES achieved the highest procyanidin yield (32.5 mg/g), while Glc and LA in a 1:5 ratio with 20 mL water/100 mL resulted in the highest anthocyanin extraction (1.58 mg/g), outperforming traditional 75% ethanol extraction method [76]. 

The different DES proved effective in extracting bioactive compounds from strawberry and raspberry by-products, yielding high-purity phenolic compounds and significant antioxidant activity comparable to acetone extraction. The highest yield and purity of phenolic compounds in the extracts were obtained with acetone (11.5%) and (36.7%) with ChCl: GlyA: Ox.A, 1:1.7:0.3. The greatest yields of total sugars and acid sugars were achieved from raspberry extrudate using acetone and from strawberry extrudate using DES-7, 82.23 and 1.88 mg/g of extract, respectively [77].

New green extraction methods for polyphenols from sour cherry pomace were also developed using NADES composed of ChCl and different hydrogen bond donors. Among these, microwave-assisted NADES preparation was the fastest method, taking less than 30 s. All NADES systems showed high efficiency for anthocyanin extraction, with choline chloride:MA being the most effective, surpassing conventional solvents by 62.33% in anthocyanin extraction efficiency [78].

Finally, NADES combined with high-speed homogenization and cavitation-burst extraction (HSH-CBE) was used to extract anthocyanins from fresh mulberries. Optimal conditions were achieved with a chloride:CA:Glc in a ratio of 1:1:1, 30% water content, and a total extraction time of 30 min. This method yielded 6.05 mg/g of anthocyanins, 1.24 times more than traditional organic solvent extraction, and provided higher stability, making it a sustainable and effective approach [79]. Table 1 presents the application of NADES in the extraction of bioactives from waste and by-products. Another study optimized the use of ChCl:OxA with pulse-ultrasonication to extract anthocyanins from blueberry pomace, achieving a high yield of 24.27 mg C3GE/g DW under specific conditions. ChCl:OxA demonstrated superior stability and protective effects for anthocyanins compared to acidified ethanol, as confirmed by stability assays and various analytical techniques [80].

## 9. Extraction of Bioactive Compounds from Vegetable Wastes and By-Products

In recent years, the extraction of bioactive compounds from vegetable waste and by-products has gained significant attention due to increasing focus on sustainability and efficient resource utilization. Often discarded, vegetable by-products are rich in antioxidants, vitamins, and other health-promoting compounds. By utilizing NADES for extracting these bioactives, waste is minimized and valuable resources are retrieved, contributing to the development of functional foods and nutraceuticals. This method not only aligns with the principles of a circular economy but also holds the potential to transform the food industry. NADESs, in combination with techniques such as homogenate-assisted extraction (HAE), microwave-assisted extraction (MAE), ultrasound-assisted extraction (UAE), or high hydrostatic pressure-assisted extraction (HHPAE), have been successfully applied to recover phenolic compounds from vegetable by-products and waste.

For example, to identify the most efficient natural solvent for quercetin extraction, its solubility was tested in various DESs and their aqueous solutions across a temperature range of 6–75 °C. The optimal DES, a mixture of ChCl and U, was then used to extract quercetin from onion tunic waste. The solubility of quercetin in pure ChCl:U at 25, 50, and 75 °C was in the range from 2.5 to 2.7 mg/mL. While ethanol extracted the largest quantity of quercetin, the ChCl:U mixture provided the purest form with the least impurities, making it highly suitable for selective extraction [81].

Further research explored the efficacy of DES, assisted by ultrasound, for extracting polyphenolic compounds from tomato pomace. The study identified phenolic acids and flavanols, with chlorogenic acid as the main compound. The extracts showed high antioxidant activity, which was confirmed through various assays and supported by theoretical studies using quantum chemistry and molecular modeling [82]. Table 2 presents the application of NADES in the extraction of bioactives from waste and byproducts.

NADESs have shown great potential for the extraction and biotransformation of bioactive compounds from various vegetable wastes and by-products. For instance, a one-pot extraction and biotransformation process using NADES was applied to pigmented rice bran. The most effective NADES, based on ChCl and xylitol, produced extracts that significantly enhanced lipid oxidative stability in oil-in-water emulsions. These enriched extracts, yielding 1525 mg eq gallic acid/100 g of rice brain, demonstrated significant antioxidant activity, suggesting their potential as natural additives [83].

ChCl -CA, -LA, -MA, and -Gly and an addition of 20% *v*/*v* water in the NADES solutions combined with homogenate—(HAE)-, microwave—(MAE)-, ultrasound—(UAE)-, or high hydrostatic pressure—(HHPAE)-assisted extractions were first successfully applied to phenolic compounds recovery from olive pomace. DES-CA and DES-LA showed the best extraction efficiency in terms of the total phenolic content and antioxidant activity. HAE proved to be the best method, with extraction efficiency superior to MAE, UAE, and HHPAE. These values ranged from 45.69 to 15.67 g d.w./g DPPH; the highest antioxidant activity was obtained for the ChCl -LA extract at 600 MPa for 10 min, followed by ChCl -MA > ChCl-CA > ChCl -Gly [84].

NADES combined with homogenate (HAE), microwave (MAE), ultrasound (UAE), and high hydrostatic pressure (HHPAE) extractions were used to recover phenolics from olive pomace. DES-CA and DES-LA had the best extraction efficiency, with HAE proving superior to other methods. HPLC showed NADES extracts were richer in phenolics than conventional solvents, supporting their use for sustainable industrial applications [85].

In another study, UAE with a choline chloride–acetic acid mixture was optimized for extracting phenolic compounds from bitter melon leaves. This method significantly improved yields compared to water, ethanol, and methanol extractions, with vanillic acid identified as the most bioaccessible phenolic compound under optimal extraction conditions (1:4.35 choline chloride–acetic acid, 20.68% water, 75 °C, 21.23 min) [86].

UAE combined with ChCl:U (1:2, 5% water content) also proved to be an effective green method for extracting solanesol from tobacco leaves, yielding significantly higher content than traditional methods. This obtained extract was characterized by FT-IR and SEM analyses, demonstrating its potential for applications in the food and pharmaceutical industries [87].

Additionally, a green extraction approach was proposed for recovering lycopene from tomatoes using hydrophobic natural deep eutectic solvents (hNADESs) based on terpenes and fatty acids. Characterized by density, rheological properties, and FT-IR spectroscopy and optimized using response surface methodology (RSM), the combination of decanoic and doC10s showed an extraction capacity comparable to acetone, making the carotenoid-rich extracts suitable for industrial food applications [88].

Another study explored the use of DES composed of magnesium chloride hexahydrate [MgCl_2_·6H_2_O] and U in different proportions (1:1 and 2:1) as extracting and stabilizing agents for red and violet betalains from beetroot (*Beta vulgaris* L.) waste. The betalain DES extract in the 2:1 ratio demonstrated superior stability, maintaining 75% of its initial content for 340 days in amber vessels, compared to the rapid degradation observed in water-based samples [89].

The hNADESs composed of Me and fatty acids, particularly a 2:1 mixture of Me and hexanoic acid, were also found to be highly efficient in extracting carotenoids from tomato industry waste (94.5 ± 3.3 μg CtE/g dm). Antiradical activity (63.7 ± 4 μmol AAE/g dm) and reducing power (26.7 ± 1.8 μmol AAE/g dm) for obtained NADES were recorded. The optimized extraction conditions significantly improved yields, highlighting hNADESs as promising sustainable solvents for carotenoid extraction with potential applications in various industries [90].

Further work focused on extracting lycopene from tomato skins using a green method that combined UAE with volatile natural deep eutectic solvent (vNADES) consisting of Me and Ty in a 1:1 ratio. While the highest lycopene concentration was obtained with n-hexane (735.9 mg/g), terpene-based solvents like α-pinene and Me:Ty DES yielded between 358.7 and 484.2 mg/g, offering a more sustainable alternative [91].

In the extraction of curcuminoids, CA and Glu (1/1) was a tailor-made solvent system to provide the highest extraction efficiency for pigments, namely bis-demethoxycurcumin at 16.54 mg/g, demethoxycurcumin at 15.12 mg/g, and curcumin at 21.18 mg/g. The study revealed that the extracted curcuminoids possessed strong antioxidant activity, good stability in different solvents, and could be efficiently recycled using solid phase extraction, emphasizing the method’s eco-friendliness and sustainability [92].

Moreover, ChCl and betaine-based natural deep eutectic solvents (NADESs) were optimized for extracting quercetin from onion and broccoli using UAE and HPLC, achieving recoveries of 88.91–98.99%. These tailor-made NADESs proved to be sustainable and safe extraction media for quercetin, isorhamnetin, and kaempferol, offering an effective alternative for biochemical applications [93]. Another study identified an efficient NADES using ChCl and 1,2-propylene glycol (ChCl-PG) at a molar ratio of 1:2, coupled with ultrasonication, for extracting phenolic compounds from broccoli leaves. The extracted phenolics, particularly neochlorogenic acid, exhibited higher total phenolic content, greater antioxidant activity, and inhibitory effects against *S. aureus*, *E. coli*, and *Salmonella* [94].

Furthermore, an efficient ultrasound and microwave-assisted ternary deep eutectic solvent (TDES) pretreatment method was developed to deconstruct the lignocellulosic structure of garlic skin and green onion root. This method achieved lignin removal rates of 90.14% and 92.34%, respectively, significantly increasing cellulose content and enhancing enzymatic hydrolysis. The effectiveness of this method in lignocellulose fractionation and cellulose digestibility was confirmed through FT-IR, XRD, and SEM analyses [95].

## 10. Circular Economy Concept Supported by Green Extraction of Bioactives

The extraction of bioactives from fruit and vegetable waste using NADES supports the circular economy by converting agricultural by-products into high-value ingredients for functional foods, nutraceuticals, and cosmetics. This process not only reduces waste but also promotes sustainability. Beyond the immediate environmental benefits, the use of NADES in bioactive extraction contributes to long-term sustainability. Traditional waste management practices, such as landfilling or incineration, often contribute to pollution and greenhouse gas emissions. By repurposing agricultural waste, the NADES extraction process helps mitigate these environmental issues, fostering a more sustainable approach to waste management. Additionally, this process reduces the need for synthetic additives in food and pharmaceutical products, further decreasing the environmental footprint of these industries.

The economic advantages of this approach are also significant. By creating new revenue streams from waste materials, this method provides economic incentives for farmers and food processors, encouraging more sustainable agricultural practices. It also supports the development of innovative products that meet the growing consumer demand for natural and health-promoting ingredients. This not only enhances market competitiveness but also drives investment in green technologies and sustainable practices.

The integration of NADES in the extraction of bioactives from fruit and vegetable waste represents a holistic approach to resource management. It aligns with the circular economy’s goals of waste reduction, resource optimization, and the creation of sustainable products, fostering a more resilient and eco-friendly industrial ecosystem. By closing the loop on waste and resource use, this innovative process contributes to a sustainable future where economic growth and environmental management are closely aligned.

## 11. Challenges and Future Directions

While current research highlights the effectiveness of NADESs in extracting bioactives from various food and agricultural by-products, several challenges remain. One of the main issues is the scalability of NADES-based extraction processes for industrial applications, which requires further investigation. Additionally, the cost-effectiveness of NADESs compared to conventional solvents needs a thorough economic analysis. Furthermore, regulatory approval and safety evaluations are critical for the widespread adoption of NADESs in the food and pharmaceutical industries.

Future research should focus on developing cost-effective and scalable extraction technologies, conducting comprehensive safety evaluations, and exploring novel NADES formulations tailored for specific pigments. Integrating NADES-based extraction with downstream processing and valorization strategies could further enhance the sustainability and economic feasibility of utilizing food and agricultural by-products.

While traditional organic solvents allow for easy concentration and purification of extracts using methods like rotary evaporation, extracting biologically active substances with NADES presents additional challenges. The efficiency of extraction can vary based on the components used, complicating the concentration process. Additionally, organic solvents benefit from established recycling and purification methods, whereas the purification and reuse of NADESs can be problematic due to potential changes in their composition during extraction. Addressing these issues is crucial for advancing NADESs in sustainable extraction. Future research should focus on optimizing extraction parameters, developing effective purification methods, and improving the recyclability of NADESs for broader industrial applications.

In conclusion, the application of NADES for extracting bioactives from waste and by-products represents a significant step towards sustainable and efficient utilization of these valuable resources. By optimizing the extraction process and addressing current challenges, NADES holds the potential to transform the extraction industry, contributing to the development of high-value bioactive products with minimal environmental impact.

## Figures and Tables

**Table 1:** Application of NADES extraction of bioactives from fruit waste and by-products.

	Natural Source	Extraction Technique	NADES	Analytes	References
1.	Citrus pomace with and without pectin	Enzymatic extraction	B-LA = 1:1ChC-CA = 2:1, ChCl-LA = 1:2, ChCl-Mal = 1:2, ChCl-Gly = 1:2, B-Gly = 1:2, ChCl-OA = 1:2	Flavonoids	[56]
2.	Pomegranate peels	UAE	ChCl with different HBDs in a 1:1 ratio: Suc, Gly, LA, CA, and Glu	Phenolic compounds	[57]
3.	Blueberry leaves	UAE	CA:Glu:water = 1:2:7.5, LA:Glu:water = 6:1:6, LA:Gln:water = 3:1:3, LA:NaAce:water = 3:1:2, ChCl:U = 1:2, ChCl:U:water = 1:2:1, ChCl: Gly = 1:2, ChCl: Gly:water = 1:2:1, ChCl:EG = 1:2, ChCl:EG:water = 1:2:1, ChCl:LA = 1:2, ChCl:OA = 1:1, ChCl:1,4-BD = 1:6, B:Gly:water = 1:2:1, B: EG:water = 1:2:1, Pro:LA = 1:1, Pro:OA = 1:1, Pro:Gly = 2:5	Phenolic compounds	[58]
4.	Orange by-products	Magnetic stirring and heating	ChCl:Glu = 2:1, ChCl:Fru = 1.9:1, ChCl:Xyl = 2:1, ChCl:Gly = 1:2, ChCl:MA = 1:1, ChCl:TA = 2:1, ChCl:LA = 1:3, ChCl:CA = 2:1, ChCl:Pro:MA = 1:1:1, LA:Glu = 5:1, MA:Glu = 1:1, Pro:MA = 1:1, B:CA = 1:1, B:MA = 1:1	Phenolic compounds	[59]
5.	Mandarin peels	Stirring at a temperature of 50 °C for 30 min.	ChCl:Acm = 1:2, ChCl: B˗1,4˗D = 1:2, ChCl:CA = 1:1, ChCl:EG = 1:1, ChCl:Gly = 1:2, ChCl:LA = 1:1, ChCl:LVA = 1:1, ChCl:MLA = 1:1, ChCl:MA = 1:1, ChCl: NMU = 1:3, ChCl:OA = 1:1, ChCl:Sol = 1:1, ChCl:ThU = 1:1, ChCl:U = 1:1, ChCl:Xyl = 1:1	Hesperidin	[60]
6.	Dried lemon peel wastes	Stirring at a temperature of 50 °C for 30 min.	Gly: ChCl = 3:1	Phenolic compounds	[61]
7.	Apple pomace	Stirring at a temperature of 50 °C for 30 min.	ChCl:OA = 1:1, ChCl: EG = 1:4	Bioactive compounds	[62]
8.	Noni-processing wastes	Pulsed electric field	ChCl:OA = 1:1, ChCl:MA = 1:1, ChCl:CA = 1:1, ChCl:LVA = 1:2, ChCl:Glz = 1:2, ChCl:TEG = 1:4, ChCl:EG = 1:2, ChCl:D-Sol = 1:1, ChCl:U = 1:2, ChCl:Glu:water = 5:2:5, ChCl:Suc:water = 5:2:5, ChCl:Fru:water = 5:2:5, B:OA = 1:1, B:TEG = 1:4, B = EG = 1:2, Pro:OA = 1:1, Pro:TEG = 1:4, Pro:EG = 1:2	Flavonoids and preparing flavonoid aglycones	[63]
9.	Noni fruit pomace	Pulsed electric field	ChCl:OxA = 1:1	Rutin and quercetin	[64]
10.	Pomegranate seeds	High-voltage electrical discharges	ChCl was employed as hydrogen bond acceptor (HBA) and CA, acetic acid (AA), LA, Gly and Glu were used as hydrogen bond donors (HBDs). HBA and HBD were mixed in a 1:1 ratio	Proteins and polyphenols	[65]
12.	Grapefruit peels	High-voltage electrical discharges	LA: ChCl = 3:1, LA:NaAce = 3:1, LA:Gln = 3:1, LA:NH_4_Ace = 3:1, ChCl:TA = 2:1, LA:Glu = 5:1	Polyphenols	[66]
11.	Mango (*Mangifera indica* L.) peel	Microwave-assisted extraction (MAE)	ChCl:U = 1:2, ChCl:Sol = 3:1, ChCl:Suc = 1:1, ChCl:Gly = 1:3, NaAce:Gly = 1:3, ChCl:LA = 1:3, NaAce:LA = 1:1, 1:2, 1:3, and 1:4, ChCl:MA = 1.5:1	Polyphenolic antioxidants	[67]
13	Orange peel waste	Stabilizing effect on the hydrolytic enzymes	ChCl:Glu = 1:1, ChCl:Gly = 1:2, ChCl:EG = 1:2, Glu:Fru:Suce = 1:1:1, Glu:EG = 1:2, sol: EG = 1:2, Glu: Gly = 1:2	Bioactive compounds	[68]
14.	Grape pomace	UMAE	ChCl:CA = 2:1, ChCl:MA = 1:1, ChCl: Pro:MA = 1:1:1, Pro:MA = 1:1, B:MA = 1:1, B:CA = 1:1, MA:Glu:Gly = 1:1:1, MA:Glu = 1:1	Anthocyanins	[69]
15.	Grape pomace	Stirring at a temperature of 50 °C for 30 min	B:CA = 1:1, B:EG = 1:2, and B:U = 1:2 were prepared by mixing betaine as HBA and three different HBDs at the appropriate stoichiometric ratio	Anthocyanins	[70]
16.	Grape pomace	Solid–liquid extraction	B:Gly = 1:2, Ch:Gly = 1:2, B:EG = 1:2, Ch:EG = 1:2, Ch:U = 1:2, B:Xyl = 1:1, Ch:Xyl = 2:1, EG:Xyl = 2:1, Me:C8 = 1:1, Ty:C8 = 1:3, Me:C10 = 1:1, Ty:C10 = 1:1, B:Scu = 1:1, Ch:Scu = 2:1, CA:Scu = 1:1, MA:Scu = 1:1, B:CA = 1:1, B:MA = 1:1, Ch:CA = 1:1, Ch:MA = 1:1, Ch:OxA = 1:1, Pro:MA = 1:1, EG:Sol2:1, B:Glc = 1:1, Ch:Glc = 1:1, CA:Glc = 1:1, MA:Gl = 1:1, EG:Glc = 2:1, Gly:Glc = 2:1, Ch:Fru = 1:1, CA:Fru = 1:1, MA:Fru = 1:1, EG:Fru = 2:1, Glc:Fru = 1:1, Ch:Sor = 1:1, CA:Sor = 2:3, EG:Sor = 2:1, Me:Cam = 1:1, Me:SA = 4:1, Me:Ty = 3:2, Ty:Cou = 3:2, Ch:Xyol = 5:2, Ch:Sol = 1:1	Polyphenols	[71]
17.	Grape seeds and skin	UAE	ChCl:CA = 2:1, ChCl:Glu = 1:1	Polyphenols	[72]
18.	Mangosteen peels	Stirring at a temperature of 50 °C for 30 min	ChCl:Gly = 1:2, ChCl:EG = 1:2, ChCl:U = 1:2, ChCl:Sol = 1:1, ChCl:LA = 1:2, ChCl:CA = 2:1, ChCl:FA = 1:2	Polyphenols	[73]
19.	Pomegranate peels	Infrared-assisted extraction and UAE	LA:ChCl = 3:1, MA:Suc = 1:1, Gly:Gln = 3:1, ChCl:Fru = 1.9:1, Glu:TA = 1:1, Gly:U = 1:1, MA:Glu:Gly = 1:1:1, LA:Gly = 3:1	Polyphenols	[74]
20.	Cranberry pomace	UAE	ChCl:LVA:EG = 1:1:2, ChCl:B:LVA = 1:1:2, ChCl: P-1,2-D:LA = 1:1:2, ChCl:LA = 1:2, ChCl: B˗1,4˗D = 1:4, ChCl:B:EG = 1:1:2, ChCl:Pro:MA = 1:1:1, ChCl:Gly = 1:2, Glu:LA = 1:5, CA:mal = 4:1	polyphenols	[75]
21.	Strawberry and raspberry waste	Stirring	ChCl:Gly = 1:2, ChCl:Suc = 1:2, ChCl: B˗1,4˗D = 1:5, ChCl: P-1,2-D = 1:1, B:Suc = 2:1, B:LVA = 1:2, ChCl: GlyA OA = 1:1.7	Bioactives	[76]
22.	Sour cherry pomace	Ultrafast MAE	ChCl:MA = 1:1, ChCl:U = 1:1, ChCl:Fru = 1:1	Polyphenols	[77]
26.	Mulberry	HSH and CBE	ChCl:CA:Glu = 1:1:1	Anthocyanin	[78]
25.	Blueberry pomace	Pulse-ultrasonication assisted extraction	ChCl:MA = 3:2, ChCl:OA = 1:1, ChCl:LA = 1:1, ChCl:CA = 1:1, ChCl:SA = 1:1, ChCl:TA = 2:1, ChCl: PG = 1:2, ChCl:Gly = 1:2, ChCl:Bnd = 2:2, ChCl:Mal = 4:1, ChCl:Glu = 1:1, ChCl:Suc = 1:1	Anthocyanin	[79]

**Table 2:** Application of NADES extraction of bioactives from vegetable waste and by-products.

No.	Vegetable Waste and By-Products	Extraction Technique	NADES Composition	Target Analyte	Reference
1.	Waste onion	Stirring with a magnetic stirrer	ChCl:U = 1:2, ChCl:Gly = 1:2, CA:Glu = 1:1, CA:Fru = 1:1, Le:U = 1:2, Le:Gly = 1:2.	Quercetin	[80]
2.	Tomato pomace	UAE	ChCl:1,2- P-1,2-D = 1:2, ChCl:LA = 1:2	Phenolic compounds	[81]
3.	Rice bran	One-pot extraction	ChCl: P-1,2-D: water = 1:1:1, ChCl:LA = 1:10, ChCl:Xyl:water = 1:1:1	Phenolics	[82]
4.	*Beta vulgaris* L. var. rubra waste	MAE	ChCl:Fru:water = 2:5:5, ChCl:Gly = 1:2, ChCl:CA:water = 2:1:6, ChCl:U = 1:2, B:Fru:water = 1:1:5, B:Gly = 1:2, B:CA:water = 2:1:6, B:U = 1:2	Bioactives	[83]
5.	Olive pomace	HAE, MAE, UAE, or HHPAE	ChCl:CA, ChCl:LA, ChCl:Mal, ChCl:Gly, all in 1:2 ratio	Phenolic compounds	[84]
6.	Bitter melon (*Momordica charantia*)	UAE	ChCl: Ace = 1:4.35	Phenolic compounds (including gallic acid, chlorogenic acid, vanillic acid, epicatechin, and quercetin-3-glucoside)	[85]
7.	Tobacco leaves	UAE	ChCl:U = 1:2		[86]
8.	Tomato	Heated at 50 °C upon stirring (750 rpm)	Men: CapA, Me: LauA, Thy:CapA, Thy: LauA, CapA: LauA in ratios 1:1, 1:2, and 2:1	Lycopene	[87]
9.	Beetroot (*Beta vulgaris*)	Heated at 50 °C upon stirring (750 rpm)	MgCl_2_·6H_2_O and U in 1:1 and 2:1 ratios	Betalains	[88]
10.	Tomato by-products	Solvent:solid 25:1, 90 min, 50 °C	Me: HeA = 2:1	Carotenoids	[89]
11.	Tomato skin	UAE	Me:Thy = 1:1	Lycopene	[90]
12.	*Curcuma longa* L	Constant stirring for 40 min	CA:Glu, MA:Glu, LA:Glu, all in 1:1 ratio	Curcumin	[91]
13.	Onion and broccoli	UAE solid–liquid method	(Ery, Rib, Xyl, fuc, ChCl–Glul, Man, Gala, ChCl–Mal, and ChCl–GluA), and B-based NADESs with (B–Ery, rib, xyl, B–Fuc, B–Glu, B–Man, B–Gal, B–Mal, and B–GluA)	Quercetin	[92]
14.	Waste broccoli leaves	UAE solid–liquid method	ChCl:MA = 1.5:1, ChCl:LA = 1:1, ChCl:Glu = 2:1, ChCl:OA = 1:1, ChCl: P-1,2-D = 1:2, ChCl: 1,3-B = 1:2, ChCl:Gly = 1:2, ChCl:CA = 3:1, ChCl:D-Sol = 1:1, ChCl:U = 1:2	Phenolic compounds	[93]
15.	Vegetable wastes	UAE and MAE	ChCl and Gly were prepared by stirring (200 rpm) the mixture of ChCl and Gly (mole ratio1:1, 1:2, 2:1)	Lignin	[94]

LA—Lactic acid; CA—citric acid; MA—malic acid; OxA—oxalic acid; Gly—glycerol; EG—ethylene glycol; U—urea; Xyl—xylose; Glc—glucose; Fru—fructose; Sor—sorbose; Cam—camphour; SA—salicylic acid; C8—octanoic acid; C10—decanoic acid; C18:2—linoleic acid; Ty—thymol; Cou—coumarin; Scu—sucrose; Xyol—xylitol; Sol—sorbitol; B—betaine; ChCl—choline chloride; Me—menthol; Pro—prolin; PDA—propanedioic acid; TA—tartaric acid; Gln—Glycine; NaAce—Na acetate; BD—butanediol; Acm—Acetamide; B-1,4-D—Butane-1,4-diol; P-1,2-D—1,2-propanediol; LVA—levulinic acid; MLA—malonic acid; NMU—N-methyl urea; THU—thiourea; TEG—triethylene glycol; FA—formic acid; Suc—sucrose; Mal—maltose; GlyA—glycolic acid; OA—oxalic acid; Bnd—butanediol; PG—propylene glycol; SA—sorbic acid; Le—lecithin; Ace—acetic acid; Men—menthol; LauA—lauric acid; CapA—capric acid; HeA—hexanoic acid; Rib—ribose; Xyl—xylose; Gala—Galactose; P-1,2-D – propanediol; 1,3- B-butanediol.

## Data Availability

The data presented in this study are available on request from the corresponding author.
